# A Modified Design for Posterior Inlay-Retained Fixed Dental Prosthesis

**DOI:** 10.1155/2015/576820

**Published:** 2015-02-16

**Authors:** Abdulaziz Samran, Mohammad Zakaria Nassani, Marwan Aswad, Amid Abdulkarim

**Affiliations:** ^1^Department of Fixed Prosthodontics, Faculty of Dentistry, University of Ibb, Ibb, Yemen; ^2^Restorative Dental Sciences Department, Al-Farabi Colleges, Riyadh, Saudi Arabia; ^3^Department of Removable Prosthodontics, Faculty of Dentistry, University of Aleppo, Aleppo, Syria; ^4^Department of Fixed Prosthodontics, Faculty of Dentistry, University of Aleppo, Aleppo, Syria

## Abstract

The aim of this paper is to report a clinical case with bilateral missing mandibular second premolars that were restored by metal-ceramic inlay-retained fixed dental prostheses. The first prosthesis was of a traditional design and the second was of a modified design. The suggested design is created by modifying the retainer wings of the traditional resin-bonded inlay-retained fixed dental prosthesis and covering the wings with composite resin. The modified design is relatively conservative, esthetic and provides an extra element for the retention of posterior metal-ceramic inlay-retained fixed dental prostheses.

## 1. Introduction

When a decision is made to replace a missing single posterior tooth in bounded saddle area by a fixed dental prosthesis (FDP), a number of prosthesis designs is available. These include traditional full veneer FDP, inlay-retained fixed dental prosthesis (IRFDP), and implant-supported FDP. A major disadvantage of full veneer FDP is the removal of significant amount of sound tooth structure of the abutment teeth. Furthermore, crown preparation may be considered a risk factor for pulp vitality or may lead to harmful pulpal reactions in the long term [1]. It has also been estimated that between 63% and 73% of the coronal tooth structure is removed when teeth are prepared for all-ceramic crowns [2]. Although the implant-supported FDPs far surpass the tooth-supported FDPs from both satisfaction and biological points of view [3], patients may express reservations about this treatment option [4]. This may be due to the relatively higher cost of implant treatment and/or patient reluctance to undertake surgical intervention. The increased awareness of oral and dental health among modern societies has led to increased popularity of the IRFDPs for the replacement of missing posterior teeth. This may be attributed to the relatively conservative nature of such dental restorations [5]. On the other hand, the IRFDP can be easily bonded to the adjacent minimally prepared abutment teeth with acceptable level of short-term survival rate [6, 7]. When treatment with dental implants is not desirable or contraindicated for a patient with missing posterior tooth in bounded saddle areas, restoration of the existing dental space by an IRFDP would be a more favorable treatment option than the traditional full veneer FDPs, provided that sufficient sound tooth structure is available in the neighboring abutments. The traditional design of IRFDP includes a pontic with mesial and distal inlay wings as retainers. The retainers take the form of inlay restorations that occupy the whole depth and width of almost class II cavities which are prepared in the adjacent abutment teeth. IRFDPs can be constructed from metal-ceramic, all-ceramic, zirconia, or fiber-reinforced composite materials. The aim of this paper is to report a clinical case with missing single posterior tooth that was restored by a modified design of metal-ceramic IRFDP.

## 2. Case Presentation

A healthy 24-year-old male patient presented at the Department of Fixed Prosthodontics, Faculty of Dentistry, University of Aleppo, Syria, with a chief concern of dental spaces. The intraoral examination revealed missing right and left mandibular second premolar teeth. The edentulous space in both sides was equal to the typical size of a mandibular second premolar. Probing depth around the adjacent teeth was within the physiological range with no pathological mobility. All abutment teeth were vital and had no apparent periodontal disease. Occlusion was analyzed preoperatively, both clinically and with the aid of mounted study models. The maxillary first molars were in class I relationship on both sides. Radiological evaluation showed that bone levels of the abutment teeth corresponded to the upper third of the root length, with no signs of active bone resorption or periapical pathology. Because treatment with implants was the best treatment option for this case, this option was explained to the patient. However, the patient refused treatment with implants and chose the IRFDP. It was planned to restore the right side of the mouth by a traditional metal-ceramic IRFDP and the left side by a modified design of metal-ceramic IRFDP. Patient consent on the proposed treatment plan was obtained. While restoration of the right mandibular dental space was carried out in accordance with the principles of treatment with traditional IRFDP, this case report will describe the different steps that were carried out for the restoration of the left mandibular dental space by a modified design of metal-ceramic IRFDP.

## 3. Prosthodontic Procedures for the Modified Metal-Ceramic IRFDP

**Figure 1:**
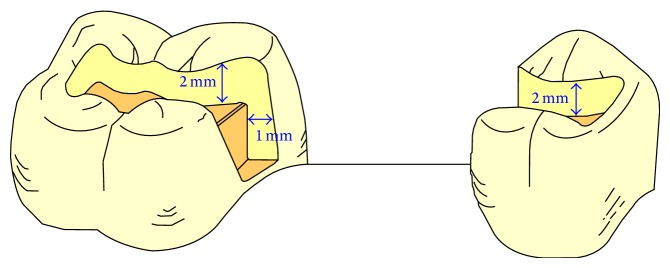
The ideal shape of the prepared cavities to receive the modified metal-ceramic IRFPD.

**Figure 2:**
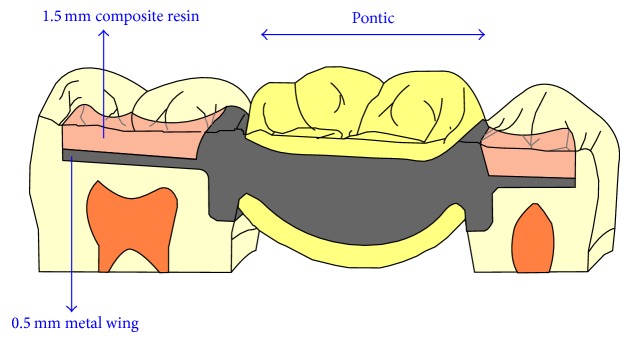
A cross sectional lingual view of the of the modified metal-ceramic IRFDP and its relation to the supporting dental structures.

**Figure 3:**
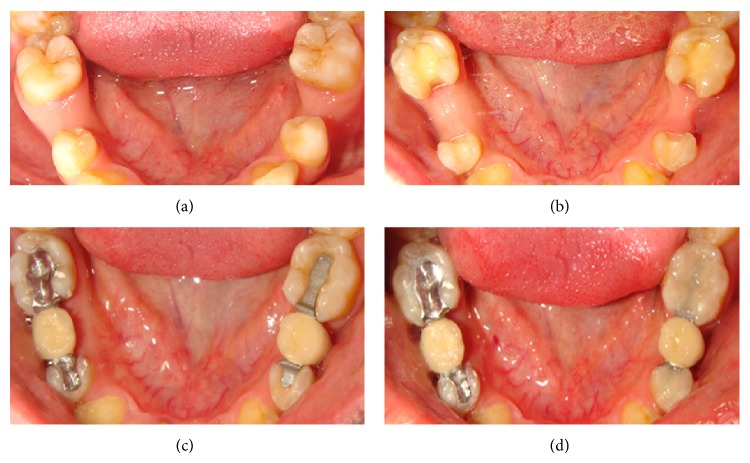
(a) Preoperative intraoral view illustrating loss of mandibular second premolars in a 24-year-old male patient. (b) Intraoral occlusal view illustrating the abutment teeth prepared to receive the metal-ceramic IRFDPs: (left) the modified design and (right) the traditional design. (c) Try-in of the IRFDPs before cementation: (left) the modified design and (right) the traditional design. (d) Intraoral occlusal view of the two IRFDPs after cementation: (left) the modified design and (right) the traditional design.

**Figure 4:**
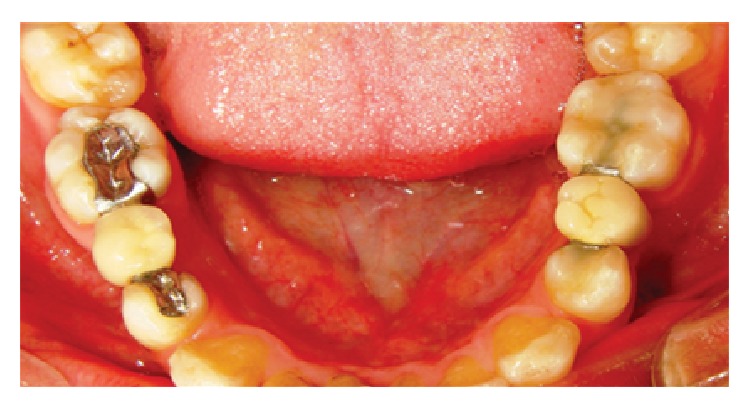
Intraoral occlusal view of the two metal-ceramic IRFDPs after 1-year follow-up time: (left) the modified design and (right) the traditional design.

The aim of the first step of the prosthodontic intervention was to prepare the abutment teeth to receive the planned IRFDP. This was performed in accordance with the general principles for preparation of inlay restorations. Almost a class II distal occlusal cavity was prepared in the mandibular left first premolar and a class II mesial occlusal cavity was prepared in the first molar. However, the preparation form was unretentive in the occlusal direction with the divergence angle of the cavity walls approximately 6–10°. Fine diamond burs were used for cavity preparation (Meisenger; Hager & Meisenger GmbH). The dimensions of the prepared cavities were as follows: the occlusal depth is approximately 2 mm, the buccolingual width is about 1/3 to 1/2 of the intercuspal distance, and the depth of the proximal box is 1 mm (1 mm shoulder with rounded internal angles and no bevels were made). All preparations were finished by rounding sharp angles.  Figure 1 shows the shape of the prepared cavities to receive the modified metal-ceramic IRFDP. The final full-arch impression was made with a combination of heavy- and light-viscosity polyvinylsiloxane (Elite HD; Zhermack). An impression of the opposing dentition was also made with irreversible hydrocolloid alginate (Hydrogum; Zhermack). An interocclusal record at maximum intercuspation position was obtained and the shade was selected with a shade guide (Vitapan 3D Master; Vita). The impressions were poured with extra hard stone and the framework of the modified IRFDP was waxed up. The wax models were invested (Sherafina; Shera GmbH) and then casted with base metal alloys (Wirbond C; Bego) with a lost-wax technique. The retainers of the modified IRFDP were designed so as not to fill the occlusal cavities with its thickness approximately 0.5 mm leaving about 1.5 mm space in the occlusal cavity. At the try-in stage, the fit and stability of the metal framework were assured. The marginal fit of the framework was checked intraorally with a silicone indicator (Fit checker; GC) and an explorer. The marginal fit was accepted when the silicone indicator paste showed a thin and homogeneous thickness. Adjustments were done if necessary. It was also assured that a minimum of 1.5 mm space occlusal to each retainer was available in the occlusal cavity. Following this stage the metal framework was sent back to the dental technician to complete its fabrication by adding porcelain to the pontic framework. A modified ridge lap design of the pontic was requested. The dental technician was instructed to make the pontic narrower at the expense of the lingual surface to avoid the formation of an uncleanable lingual surface and also was instructed to open embrasure spaces adjacent to the pontic to allow room for interproximal tissues and access for oral hygiene [8]. When the IRFDP was returned back from the dental lab it was tried in to assure appropriate fit and occlusion before the final glazing was completed by the dental lab. Luting the modified metal-ceramic IRFDP was carried out in two stages: the first stage comprised cementation of the metal framework. The inner and outer surfaces of the modified retainers were airborne-particle abraded with 50 *μ*m Al_2_O_3_ (aluminum oxide abrasive; Heraeus Kulzer GmbH) under 2.5 bar pressure. All cavities were then conditioned using a self-etching bonding material (Bond Force, Tokoyama Dental Corp., Tokyo, Japan). The outer and inner surfaces of the retainers of the modified metal-ceramic IRFDP were coated with metal primer (Metal/Zirconia Primer; Ivoclarvivadent AG). Following this stage the modified IRFDP was luted with adhesive resin cement (Bistite; Tokoyama) according to manufacturer's instructions. After the completion of the first step of cementation, excess resin cement over the retainers was removed to allow adequate space for the application of composite resin in the second step of cementation. At the second step of cementation a posterior composite resin (Estelite Σ, Tokoyama Dental Corp., Tokyo, Japan) was applied in the prepared cavities over the metal wings of the modified IRFDP. The procedure is similar to that of filling class II cavities with composite resin by layers technique and light-cure.  Figure 2 demonstrates a cross sectional lingual view of the modified metal-ceramic IRFDP and its relation to the supporting dental structures. Figures 3(a)–3(d) present clinical photographs of the reported case. At the stage of prosthesis delivery, the patient received postoperative care instructions, and recall appointments were scheduled. Within the one year observation time there was no debonding and no need for repair or retreatment other than maintenance procedures, including oral hygiene and prophylaxis. During the latest follow-up examination after one year of prosthesis delivery the patient reported that he was satisfied with both traditional and the modified metal-ceramic IRFDPs.  Figure 4 shows the reported case after a 1-year follow-up time.

## 4. Discussion

Clinical reports about the outcome of treatment with IRFDPs indicate that this treatment modality is an effective treatment option for the replacement of missing posterior teeth with up to 7 years mean survival time and debonding or fractures as main complications [9–12]. In this case report, a metal-ceramic IRFDP with a modified design was used for the restoration of a missing mandibular left second premolar bounded by intact teeth. This suggested design is indicated for a single posterior tooth replacement in bounded saddles areas [13], for example, replacement of missing second premolar, first molar, or second molar with the existence of a sound neighboring third molar tooth. However, teeth of mobility grade II or more, wide edentulous spaces, and heavily restored abutment teeth can be considered as contraindications.

The metal framework of the suggested IRFDP should be constructed to have sufficient rigidity to resist occlusal and masticatory forces in the posterior region of the mouth. To achieve this aim the thickness of the metal wings should be at least 0.5 mm. Furthermore, special attention should be paid to design adequately thick and rigid connectors.

The thickness of the composite resin layer over the metal wings should be at least 1.5 mm; this is an important measure to achieve adequate degree of polymerization of the composite resin [14] and hence to obtain sufficient rigidity to resist functional forces transmitted from the prosthesis to the underlying metal wings of the framework. Furthermore, placement of the composite resin layer over the metal wings may enhance resistance to displacement of the prosthesis retainers outwards the prepared cavities. The retention elements of the modified metal-ceramic IRFDP are the mechanical friction between the metal framework and tooth walls in the prepared cavities, the resin cement used in the first step of prosthesis cementation, and the adhesion between the composite resin placed over the metal wings and the dental tissues of the prepared cavities. With this suggested design the disadvantages of the traditional metal-ceramic IRFDP, such as visibility of the metal-retainer, change of natural tooth translucency, and partial debonding of the retainers, can be overcome. Esthetic is better compared to traditional metal-ceramic IRFDP as placement of the composite resin over the metal wings may mask the metal color. However, a greyish appearance of the abutment teeth may develop in some cases. Luting the modified IRFDP by resin cement and placement of composite resin over the retainers may reduce debonding rate and promote resistance to displacement. However, this hypothesis needs to be substantiated through laboratory and clinical research work. A possible disadvantage of such design is the potential for gradual wear of the composite resin layer placed over the wings of the IRFDP. This may require periodical follow-up visits to the dental clinic to assure the integrity of the bridge structure.

## 5. Conclusion

This paper reported the use of a modified design of metal-ceramic IRFDP in the restoration of missing posterior tooth in bounded saddle area in the mandible of a young patient. The modified design has been created by altering the retainer wings in the traditional resin-bonded metal-ceramic IRFDP and covering the wings with a composite resin restorative material. The suggested design is relatively conservative, esthetic and provides an extra element for the retention of posterior metal-ceramic IRFDP. However, clinical trials are required to examine the long-term durability of this IRFDP and to compare its performance with other fixed bridge designs. A finite element analysis to study forces acting on such IRFDP is suggested.
